# Tomatidine Represses Invasion and Migration of Human Osteosarcoma U2OS and HOS Cells by Suppression of Presenilin 1 and c-Raf–MEK–ERK Pathway

**DOI:** 10.3390/molecules25020326

**Published:** 2020-01-13

**Authors:** Min-Hong Hsieh, Jia-Sin Yang, Renn-Chia Lin, Yi-Hsien Hsieh, Shun-Fa Yang, Horng-Rong Chang, Ko-Hsiu Lu

**Affiliations:** 1 Institute of Medicine, Chung Shan Medical University, Taichung 402, Taiwan; dm239644@gmail.com (M.-H.H.); gazn_sheep@yahoo.com.tw (J.-S.Y.); ysf@csmu.edu.tw (S.-F.Y.); 2 Department of Orthopedics, Dalin Tzu Chi Hospital, Buddhist Tzu Chi Medical Foundation, Chiayi 622, Taiwan; 3 Department of Medical Research, Chung Shan Medical University Hospital, Taichung 402, Taiwan; 4 Department of Orthopedics, Chung Shan Medical University Hospital, Taichung 402, Taiwan; cshy594@csh.org.tw; 5 School of Medicine, Chung Shan Medical University, Taichung 402, Taiwan; 6 Division of Hyperbaric Oxygen Therapy and Wound Medicine, Chung Shan Medical University Hospital, Taichung 402, Taiwan; 7 Institute of Biochemistry, Microbiology and Immunology, Chung Shan Medical University, Taichung 402, Taiwan; hyhsien@csmu.edu.tw; 8 Division of Nephrology, Department of Medicine, Chung Shan Medical University Hospital, Taichung 402, Taiwan

**Keywords:** ERK, metastasis, osteosarcoma, PS-1, tomatidine

## Abstract

Osteosarcoma, which is the most prevalent malignant bone tumor, is responsible for the great majority of bone cancer-associated deaths because of its highly metastatic potential. Although tomatidine is suggested to serve as a chemosensitizer in multidrug-resistant tumors, the anti-metastatic effect of tomatidine in osteosarcoma is still unknown. Here, we tested the hypothesis that tomatidine suppresses migration and invasion, features that are associated with metastatic process in human osteosarcoma cells and also investigate its underlying pathway. Tomatidine, up to 100 μM, without cytotoxicity, inhibited the invasion and migration capabilities of human osteosarcoma U2OS and HOS cells and repressed presenilin 1 (PS-1) expression of U2OS cells. After the knockdown of PS-1, U2OS and HOS cells’ biological behaviors of cellular invasion and migratory potential were significantly reduced. While tomatidine significantly decreased the phosphorylation of c-Raf, mitogen/extracellular signal-regulated kinase (MEK), and extracellular signal-regulated protein kinase (ERK)1/2 in U2OS cells, no obvious influences on p-Jun N-terminal kinase, p38, and Akt, including their phosphorylation, were observed. In ERK 1 silencing U2 OS cells, tomatidine further enhanced the decrease of their migratory potential and invasive activities. We conclude that both PS-1 derived from U2OS and HOS cells and the c-Raf–MEK–ERK pathway contribute to cellular invasion and migration and tomatidine could inhibit the phenomenons. These findings indicate that tomatidine might be a potential candidate for anti-metastasis treatment of human osteosarcoma.

## 1. Introduction

Osteosarcoma, which mainly arises from the metaphysis of long bones, is the most prevalent malignant bone tumor with a peak of incidence at 10–15 years and the second incidence peak in older adulthood [1,2]. The poor prognosis of metastatic osteosarcoma is due to its highly metastatic potential to cause most treatment failures and high mortality rates. According to radiological staging, surgical techniques, and new chemotherapy protocols, the combination of surgery and chemotherapy for osteosarcoma has increased the long-term survival chances to approximately 68% through limb-sparing surgeries [3,4]. However, the potent metastatic transfer to the lungs is still responsible for most treatment failures and it is accountable for one of the most lethal pediatric malignancies.

Cancer metastasis involves highly coordinated, sequential, and complex pathways that are collectively termed the metastasis cascade [5,6]. These pathways include the detachment of cancer cells, epithelial-mesenchymal transition (EMT), degradation of the extracellular matrix (ECM), invasion, and migration, penetrating through the basement membrane of blood and lymph vessels, intravasation, traveling through lymph fluid and bloodstream, adhering to endothelial cells of vessels, extravasation, mesenchymal-epithelial transition, and re-establishment of growth at a distant site [7,8].

After the EMT of cancer cells, the invasion of the basement membrane proceeds through a series of discrete steps and various proteases predominantly control the degradation of the ECM and the basement membrane [9]. Of these proteases, urokinase-type plasminogen activator (u-PA), matrix metalloproteinase (MMP)-2 (gelatinase A, 72 kDa), and MMP-9 (gelatinase B, 92 kDa) are considered to be the most crucial enzymes for controlling the degradation of the main constituent of the ECM, and they are substantially involved in cancer invasion and metastasis [10,11]. Thus, suppressing MMP-2 or MMP-9-mediated cellular invasion and migration might generate a putative anti-metastasis effect.

Mitogen-activated protein kinases (MAPKs), which are a family of serine/threonine kinases, including extracellular signal–regulated kinase (ERK) 1/2, c-Jun N-terminal kinase (JNK) 1/2, and p38, are known to participate in various signaling cascades that play an important regulatory role in cell growth, differentiation, apoptosis, and metastasis [12]. Metastasis is also probably regulated by the phosphatidylinositide-3 kinase (PI3K) and Akt signaling pathway, which is involved in many cellular processes, including cell motility and cell adhesion [13,14]. The activation of MAPKs and PI3K/Akt is followed by the phosphorylation of various cytosolic substrates that participate in numerous cellular activities, such as cell proliferation, differentiation, apoptosis, angiogenesis, invasion, and migration [15]. The inhibition of MAPKs and PI3K/Akt signaling pathways might potentially prevent proliferation, angiogenesis, invasion, and metastasis in a wide range of tumors [16,17,18].

Presenilin 1 (PS-1, encoded by *PSEN1*), which is a widely presented multi-transmembrane domain protein and primarily located on the plasma membrane, endoplasmic reticulum, and Golgi apparatus, functions as a core catalytic subunit of the γ-secretase complex that is involved in the cleavage of several type-I transmembrane proteins, including the β-amyloid precursor protein, Notch, CD44, vascular endothelial growth factor receptor, E-cadherin, and *N*-cadherin [19,20,21]. PS-1 plays an exclusive role in various carcinogenesis, including cell proliferation, apoptosis, cell adhesion, and others in brain, lung, breast, skin, and gastric and colorectal cancers [22,23,24,25,26,27].

Tomatidine is an aglycone of the glycoalkaloid tomatine that is mainly found in the stems and leaves of the tomato plant [28], and its inhibition of the growth of cancer cells shows a much lesser degree than that of tomatine [29]. Tomatine molecules serve as a natural defense against plant fungi, viruses, bacteria, and insects [28,30], and they are known for their popular and powerful anti-oxidative stress abilities and radical-spread limitations. Additionally, tomatine helps to fight different types of cancer through ways, such as the inhibition of proliferation, the induction of apoptosis, and the suppression of migration and invasion in a wide variety of cancer cells [18,31,32]. After consumption, tomatine is converted to tomatidine in the intestine [33], and tomatidine probably acts as a physiologically active substance that possesses an anti-metastatic property [34]. However, the effect of tomatidine on human osteosarcoma metastasis remains unclear; hence, we investigated whether tomatidine affects the invasion and migration of human osteosarcoma cells and attempted to define its underlying mechanisms.

## 2. Results

### 2.1. Cytotoxicity of Tomatidine in Osteosarcoma U2OS and HOS Cells

**Figure 1:**
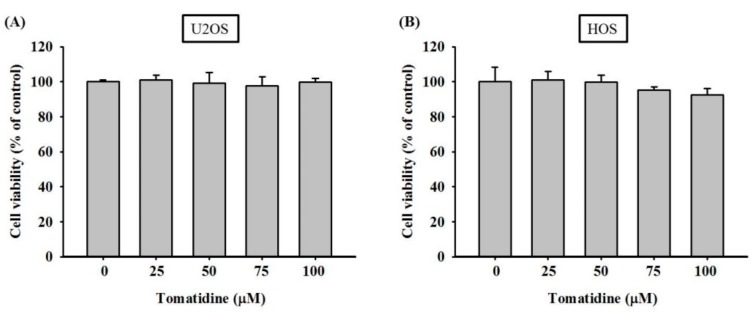
Effects of tomatidine on the cell viability of U2OS and HOS cells. (**A** and **B**) Using an Microculture Tetrazolium (MTT) assay, the effects of tomatidine on the viability of U2OS and HOS cells treated with tomatidine (0–100 μM) for 24 h were detected and illustrated after quantitative analysis. Results are shown as mean ± S.D. ANOVA analysis with Turkey’s posteriori comparison was used. (A) *n* = 3. F = 0.265, *p* = 0.894. (B) *n* = 4. F = 2.067, *p* = 0.136.

For the cell viability experiment, a microculture tetrazolium (MTT) (3-(4,5-dimethylthiazol-2-yl)-2,5-diphenyltetrazolium bromide) colorimetric assay was performed to determine the cytotoxicity of tomatidine. After 24 h of treatment, the viability of osteosarcoma U2OS and HOS cells in the presence of concentrations of 25, 50, 75, and 100 μM of tomatidine was not significantly different to that of the controls (0 μM) in MTT assay (U2OS: *p* = 0894; HOS: *p* = 0.136) (Figure 1). Thus, a 24-h treatment with tomatidine up to 100 μM had no cytotoxic effect on U2OS and HOS cells. We used this concentration range for tomatidine in all subsequent experiments to investigate its anti-metastatic properties.

### 2.2. Tomatidine Represses U2OS and HOS Cells Migration and Invasiveness

**Figure 2:**
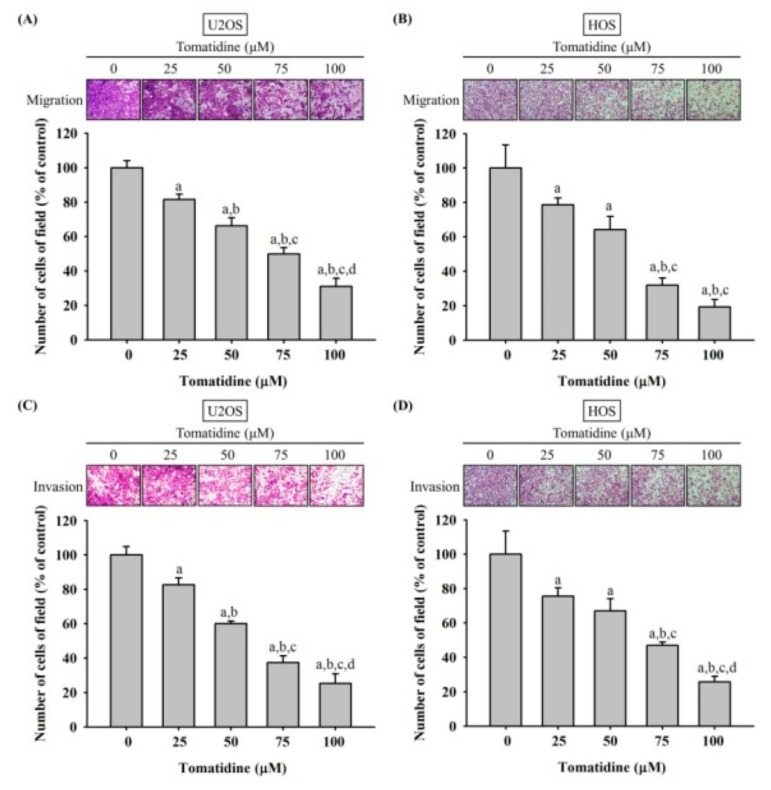
Effects of tomatidine on in vitro cellular migration and invasion of U2OS and HOS cells. Cell migration (**A** and **B**) and invasion (**C** and **D**) assays after various concentrations (0, 25, 50, 75, and 100 μM) of tomatidine treatment for 24 h in U2OS and HOS cells were measured as described in the Materials and Methods section. Results are shown as mean ± S.D. *n* = 3. ANOVA analysis with Turkey’s posteriori comparison was used. (A) U2OS: F = 125.713, *p* < 0.001; (B) HOS: F = 56.973, *p* < 0.001; (C) U2OS: F = 159.838, *p* < 0.001; (D) HOS: F = 43.987, *p* < 0.001. a Significantly different, *p* < 0.05, when compared to the control. b Significantly different, *p* < 0.05, when compared to 25 μM. c Significantly different, *p* < 0.05, when compared to 50 μM. d Significantly different, *p* < 0.05, when compared to 75 μM.

We used a modified Boyden chamber migration and invasion assays to test the effect of tomatidine on invasive properties of U2OS and HOS cells in vitro. After treating for 24 h, the Boyden chamber assay without Matrigel showed that tomatidine significantly dose-dependently reduced the migratory potential in U2OS and HOS cells (U2OS: *p* < 0.001; HOS: *p* < 0.001) (Figure 2). The modified Boyden chamber assay with Matrigel also showed that tomatidine dose-dependently reduced the invasive activity in U2OS and HOS cells (U2OS: *p* < 0.001; HOS: *p* < 0.001).

### 2.3. Tomatidine Reduces PS-1 Expression of U2OS Cells

**Figure 3:**
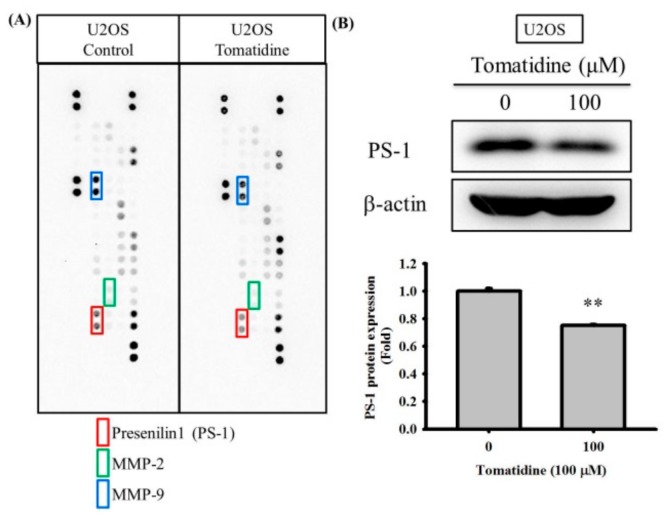
Presenilin 1 expression of tomatidine-treated in osteosarcoma U2OS cells. (**A**) The protease array after treatment with 100 μM of tomatidine for 24 h in U2OS cells were employed as described in the Materials and Methods section. (**B**) Western blot analysis after various concentrations (0 and 100 μM) of tomatidine treatment for 24 h in U2OS cells were measured as described in the Materials and Methods section and the effects were illustrated after quantitative analysis. The results are shown as mean ± S.D. *n* = 3. Student’s t-test was used. (**B**) *p* = 0.001. **Significantly different: *p* < 0.01, when compared with the control group (0 μM).

We employed the protease array, which showed repression of PS-1 secretion in U2OS cells after treatment of 100 μM tomatidine for 24 h, to identify the underlying mechanism of the anti-metastatic actions of tomatidine in osteosarcoma cells, (Figure 3A). However, no significant effects on MMP-2 and nine secretions were observed in the protease array. We subsequently performed the western blot analysis to validate the finding in the protease array and found that 100 μM of tomatidine significantly repressed the PS-1 protein expression of U2OS cells (*p* = 0.001) (Figure 3B).

### 2.4. PS-1 Knockdown Reduces Migration and Invasion of U2OS and HOS Cells

**Figure 4:**
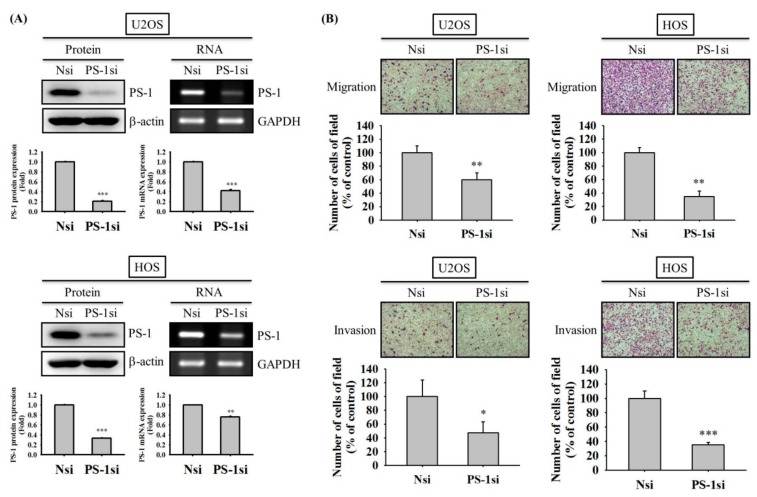
Effects of PS-1 knockdown on migration and invasion of U2OS and HOS cells. Using Reverse Transcription-Polymerase Chain Reaction (RT-PCR) to confirm siRNA directly against PS-1 expression (**A**), modified Boyden chamber assays without and with Matrigel coating after treatment of PS-1 siRNA for 48 h in (**B**) U2OS and HOS cells were conducted and the effects were illustrated after quantitative analysis. The results are shown as mean ± S.D. *n* = 3. Student’s t-test was used. (**A**) U2OS: protein: *p* < 0.001 and RNA: *p* < 0.001, HOS: protein: *p* < 0.001 and RNA: *p* = 0.002; (**B**) U2OS: migration: *p* = 0.008 and invasion: *p* = 0.034, HOS: migration: *p* = 0.001 and invasion: *p* < 0.001. *Significantly different: *p* < 0.05, **Significantly different: *p* < 0.01, ***Significantly different: *p* < 0.001, when compared with the control group (Non-siRNA). Nsi: non-small interfering RNA; PS-1si: presenilin-small interfering RNA.

We transformed cells with a small interfering RNA (siRNA) targeting PS-1 expression for 24 h and measured the protein expression and the mRNA level in western blotting and reverse transcription-polymerase chain reaction (RT-PCR), respectively, to further confirm whether reduction of PS-1 interferes with migratory potential and invasive activity of U2OS and HOS cells (U2OS: protein: *p* < 0.001 and RNA: *p* < 0.001; HOS: protein: *p* < 0.001 and RNA: *p* = 0.002) (Figure 4A). Subsequently, we performed Boyden chamber migration and modified Matrigel invasion assays while using siRNA of PS-1 for 24 h and 48 h to compare the amount of migratory and invasive cells, respectively. Unsurprisingly, the knockdown of PS-1 significantly decreased the migratory potential and invasive activities of U2OS and HOS cells (U2OS: migration: *p* = 0.008 and invasion: *p* = 0.034; HOS: migration: *p* = 0.001; and, invasion: *p* < 0.001) (Figure 4B).

### 2.5. Tomatidine Reduces the c-Raf–MEK–ERK Pathway in U2OS Cells

**Figure 5:**
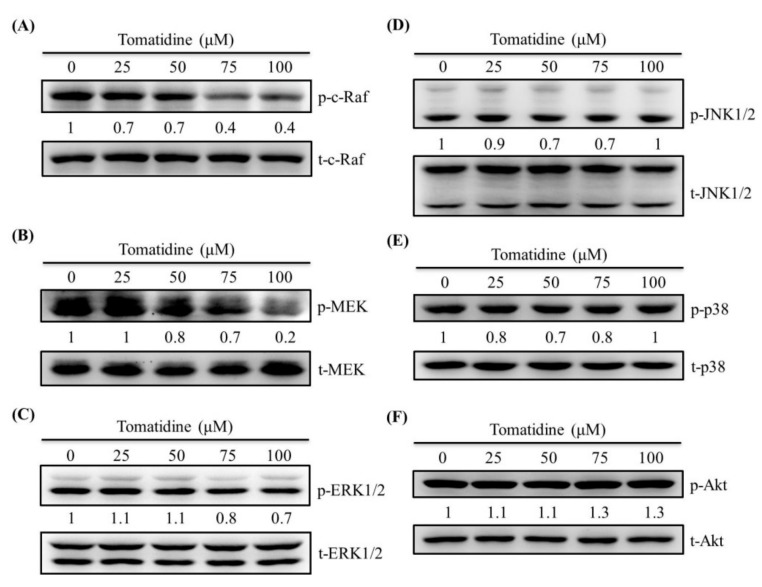
Effects of tomatidine on Raf, mitogen/extracellular signal-regulated kinase (MEK), mitogen-activated protein kinases (MAPKs), and phosphatidylinositide-3 kinase-Akt (PI3K-Akt) **in** U2OS cells. Western blot analyses for total or phosphorylated forms of (**A**) Raf, (**B**) MEK, (**C**) ERK 1/2, (**D**) JNK 1/2, (**E**) p38, and (**F**) Akt after various concentrations (0, 25, 50, 75, and 100 μM) of tomatidine treatment for 24 h in U2OS cells were measured as described in the Materials and Methods section and the effects were illustrated after quantitative analysis.

Western blotting was employed to further investigate the molecular mechanisms since MAPKs and PI3K pathways may be dependent signaling of PS-1. In the analysis, c-Raf, mitogen/extracellular signal-regulated kinase (MEK), MAPKs, and PI3K-Akt pathways were detected in U2OS cells. As a result, tomatidine decreased the phosphorylation of c-Raf, MEK, and ERK 1/2 in U2OS cells, but no obvious influence on JNK 1/2, p38, and Akt, including their phosphorylation, was observed (Figure 5).

### 2.6. Tomatidine Inhibits Cellular Migration and Invasion in ERK 1 Knockdown U2OS Cells

**Figure 6:**
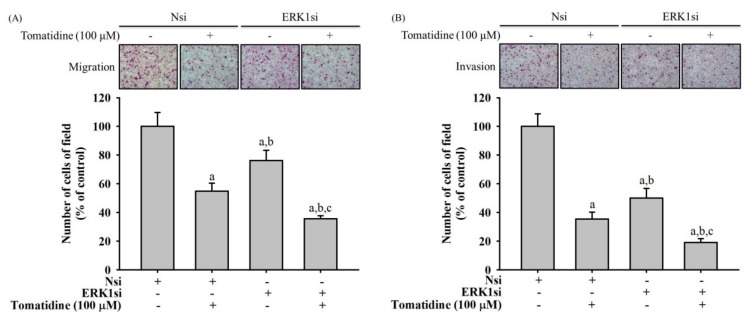
Effects of ERK 1 knockdown on biological behaviors of migration and invasion in tomatidine-treated U2OS cells. Using siRNA directly against the ERK 1 expression, modified Boyden chamber assays (**A**) without and (**B**) with Matrigel coating after treatment of PS-1 siRNA for 48 h in U2OS cells with or without treatment of 100 μM tomatidine were conducted. Results are shown as mean ± S.D. *n* = 3. ANOVA analysis with Turkey’s posteriori comparison was used. (**A**) F = 51.079, *p* < 0.001; (**B**) F = 95.285, *p* < 0.001. a) Significantly different, *p* < 0.05, when compared to control. b) Significantly different, *p* < 0.05, when compared to 100 μM of tomatidine. c) Significantly different, *p* < 0.05, when compared to siERK1.

We conducted siRNA directly against the ERK 1 with and without treatment of 100 μM tomatidine to identify whether the ERK pathway interferes with migratory potential and invasive activities in U2OS cells, and performed Boyden chamber migration and modified Matrigel invasion assays to compare the amount of migratory and invasive cells. Predictably, the knockdown of ERK 1 significantly decreased the migratory potential and invasive activities in U2OS cells (*p* < 0.05 and *p* < 0.05, respectively) and tomatidine further enhanced the decrease of migratory potential and invasive activities in ERK 1 silencing U2OS cells (*p* < 0.05 and *p* < 0.05, respectively) (Figure 6). However, with and without treatment of 100 μM tomatidine, ERK 1 knockdown could not further enhance the decrease of PS-1 expression (data not shown), which implies that the c-Raf–MEK–ERK pathway might be not the upstream signaling of PS-1.

## 3. Discussion

In the study, tomatidine, without cytotoxicity, attenuated migratory potential and invasiveness of U2OS and HOS cells. Although MMP-2 and MMP-9 are key enzymes and they contribute to the process of osteosarcoma cell invasion and metastasis in our previous research [35,36,37,38], there were no effects of tomatidine on MMP-2 and nine secretions of U2OS cells in the protease array. Intriguingly, the repression of PS-1 in U2OS cells was observed after treatment of 100 μM tomatidine and the tomatidine’s repression of PS-1 protein expression was verified in western blotting. The silencing of PS-1 confirmed the anti-metastatic properties of migration and invasion of U2OS and HOS cells by PS-1. Through a further analysis of MAPKs and the PI3K pathways, tomatidine decreased the phosphorylation of c-Raf, MEK, and ERK 1/2 in U2OS and HOS cells, whereas there was no evident influence on JNK 1/2, p38, and Akt, and their phosphorylation. Furthermore, the decrease of migratory potential and invasive activities, which was caused by the ERK 1 knockdown in U2OS cells, was enhanced by tomatidine. These results implied that tomatidine’s inhibition of invasion and migration in human osteosarcoma U2OS and HOS cells resulted from the attenuation of PS-1 and the c-Raf–MEK–ERK pathway, rather than JNK, p38, and PI3K-Akt signaling.

PS homologs PS-1 and PS-2 participate in several signaling pathways that regulate cell survival and tumorigenesis. PS-1 mutant overexpression has been reported to induce cell apoptosis [39], while the loss of PS-1 and mutant PS-1 mice have higher skin and carcinogen-induced brain tumorigenesis, respectively [24,40]. PS-1 promotes tumor invasion and metastasis of gastric cancer both in vitro and in vivo, in addition to the positive correlation with lymph node metastasis and the poor overall survival rate [25]. Conversely, the γ-secretase inhibitor DAPT inhibits gastric cancer cell growth and EMT and the results of the treatment are consistent with the outcomes of treatment with PS-1 silencing [25,26]. The therapeutic effect of γ-secretase inhibition was also observed in lung cancer by the derepression of DUSP1 and inhibition of ERK [27]. In the present study, we found that tomatidine represses PS-1 to inhibit the biological behaviors of migration and invasion in U2OS and HOS cells, which indicates that PS-1 might represent a novel prognostic biomarker and a potential therapeutic target for anti-metastasis treatment of osteosarcoma. Moreover, notch signaling regulates osteosarcoma proliferation and migration through ERK phosphorylation, so PS might be the upstream signaling of the ERK pathway and the inhibition of PS can lead to ERK activation [41]. However, in the study, the silencing of ERK 1 seemed not to affect PS-1 expression, which suggests that the c-Raf–MEK–ERK pathway might be not the upstream signaling of PS-1. While the c-Raf–MEK–ERK pathway and PS-1 pathway both simultaneously contribute to invasion and migration of U2OS and HOS cells, they might be independently or the c-Raf–MEK–ERK pathway might be the downstream signaling of PS-1. Hence, further tests are required to make it explicitly clear. Anyway, PS-1 and the c-Raf–MEK–ERK pathways both actually affect the invasion and migration of U2OS and HOS cells.

The diverse MAPK members and PI3K/Akt are activated in response to various extracellular stimuli and have distinct downstream targets, including cell motility, migration, invasion, proteinase-induction, and angiogenesis, which all contribute to metastasis [42]. Besides, ERK 1/2 and JNK are thought to play a central role in regulating the expression of MMPs to implicate cell migration and proteinase-induction [16,17,42]. Tomatine, which is a secondary metabolite from tomato, suppresses MMP-2 and MMP-9 activities and cell proliferation in breast cancer MCF-7 cell line and structure-activity relationships of α-, β_1_-, γ-, and δ-tomatine and tomatidine against various cancer cells have been studied [29,43]. Alpha-tomatine inactivates PI3K/Akt and ERK signaling pathways and nuclear factor (NF)-κB and AP-1 binding activities to inhibit the invasion and migration of human lung adenocarcinoma A549 cells by reducing u-PA MMP-2 and MMP-9 [18]. However, the invasion and migration of human non-small cell lung cancer NCI-H460 cells are suppressed by α-tomatine through inactivating the focal adhesion kinase/PI3K/Akt signaling pathway, which reduces the binding activity of nuclear factor (NF)-κB and downregulates the MMP-7 expression [44].

Of particular interest is that tomatidine inhibits iNOS and cyclooxygenase-2 expressions to display the anti-inflammatory effect through the suppression of NF-κB and JNK pathways in LPS-stimulated mouse macrophages [45]. In addition to anti-inflammatory, anti-tumorigenic, and lipid-lowering activities [45,46], tomatidine has been suggested to serve as a chemosensitizer in combination chemotherapy, which uses chemotherapeutic drugs for the treatment of multidrug-resistant cancers [47]. Moreover, tomatidine inhibits the invasion of human lung adenocarcinoma A549 cells through the suppression of ERK and Akt pathways and MMP-2 and 9 expressions [34]. However, in the study, tomatidine’s inhibitory properties of migration and invasion in U2OS and HOS cells are induced by the suppression of the c-Raf–MEK–ERK 1/2 pathway and the repression of PS-1 secretion, but that has no effect on MMP-2 and 9. These findings reveal a unique concept of pathway and direction for tomatidine in anti-metastatic therapy of osteosarcoma. In future, the determination of therapeutic potential and pharmacodynamics properties of tomatidine on osteosarcoma metastasis in vivo is imperative.

## 4. Materials and Methods 

### 4.1. Materials

Cell culture materials, including Dulbecco’s modified Eagle medium (DMEM), minimum essential medium (MEM), and fetal bovine serum (FBS) were purchased from Gibco Life Technologies (Gaithersburg, MD, USA). Antibodies that were specific for β-actin, Akt, and p38 were obtained from BD Biosciences (San Jose, CA, USA). Additionally, antibodies that were specific for phosphorylated ERK 1/2, JNK 1/2, Akt, c-Raf, and MEK, as well as ERK 1/2, JNK 1/2, c-Raf, and MEK were purchased from Cell Signaling Technology (Danvers, MA, USA). PS-1 was obtained from Abcam (Cambridge, UK). Human Protease Assay Kit was purchased from R&D Systems (Minneapolis, MN, USA).

### 4.2. Cell culture and Tomatidine Treatment 

Being obtained from the Food Industry Research and Development Institute (Hsinchu, Taiwan), the human osteosarcoma U2OS (15-yr-old female) cells and HOS (13-yr-old female) cells were supplemented with 10% FBS and 1% penicillin/streptomycin and then cultured in DMEM and Eagle’s MEM, respectively. The cell cultures were maintained at 37 °C in a humidified atmosphere of a 5% CO2 incubator. Tomatidine was purchased from Sigma-Aldrich (St. Louis, MO, USA).

### 4.3. Microculture Tetrazolium (MTT) Assay

We plated 8.5 × 10^4^/well U2OS cells and 7.5 × 10^4^/well HOS cells in 24-well plates for 16 h and then treated different concentrations (0, 25, 50, 75, and 100 μM) of tomatidine at 37 °C for 24 h. After the exposure period, MTT assay was performed, as described previously [17,48].

### 4.4. Cell Migration and Invasion Assays

After treatment with the indicated concentrations of tomatidine (0, 25, 50, 75, and 100 μM), the cells were seeded into the upper section of the Boyden chamber (Neuro Probe, Cabin John, MD, USA) without or with Matrigel at densities of 2.0 × 10^5^/mL for the U2OS cells and HOS cells, and then incubated at 37 °C for 24 h, respectively. Finally, the migratory cells in the Boyden chamber migration assay and invasive cells in the modified Boyden chamber invasion assay were counted under a light microscope, as described previously [17,48].

### 4.5. Protease Array Analysis

A protease array (35 proteases) analysis was used to evaluate the protein lysates from vehicle- or 100 μM tomatidine-treated cells, according to the manufacturer’s protocols (Human Protease Array Kit, Catalog Number ARY021B, R&D Systems, Minneapolis, MN).

### 4.6. Protein Extraction and Western Blot Analysis

The protease array results of PS-1 was confirmed and signaling pathways were detected while using western blot analysis. We plated 8.5 × 10^5^ U2OS and 7.5 × 10^5^ HOS cells in 6 cm plates for 16 h and then treated them with different concentrations (0, 25, 50, 75, and 100 μM) of tomatidine for 24 h, and the total cell lysates of U2OS and HOS cells were prepared to investigate the molecular mechanism further, as described previously [17,37,48]. Western blot analysis was performed using either specific primary antibodies against PS-1, c-Raf, MEK, three MAPKs (ERK 1/2, JNK 1/2, and p38), and Akt or with the specific antibodies for unphosphorylated or phosphorylated forms of the corresponding c-Raf, MEK, ERK 1/2, JNK 1/2, p38, and Akt. PS-1 (ab76083), and β-actin (ab8226) antibodies were purchased from Abcam (Cambridge, UK). p-c-Raf (#9427), c-Raf (#9422), p-MEK (#9121), MEK (#9122), p-ERK (#4370), ERK (#9102), p-JNK (#9251), JNK (#9258), and p-AKT (#4060) antibodies were purchased from Cell Signaling Technology (Danvers, MA, USA). p-p38 (#612281), p38 (#612168), and AKT (#610860) antibodies were purchased from BD Biosciences (San Jose, CA, USA).

As described previously, blots were then incubated with a horseradish peroxidase goat anti-rabbit or anti-mouse IgG for 1 h and the intensity of each band was measured by densitometry [17,37,48].

### 4.7. Reverse Transcription-Polymerase Chain Reaction (RT-PCR)

For RT-PCR, we plated 5 × 10^5^ U2OS cells and HOS cells in 6 cm plates for 24 h. After treating the cells with PS-1 siRNA for 72 h, the total RNA was extracted while using Total RNA mini kit (Geneaid, New Taipei City, Taiwan) and reverse transcribed into cDNA while using High Capacity cDNA Reverse Transcription kit (Applied Biosystems, CA). Procedures of complementary DNA (cDNA) synthesis and PCR amplification were performed, as described previously [17,49]. The specific primer sequences for these genes are as following: PS-1: 5′-AGATCTGAGTCCAAGAATCGCGGA-3′ (forward), 5′-AAGCTTCTACTAATCCCGGCCCAAGG-3′ (reverse), and GAPDH: 5′-CGGAGTCAACGGATTTGGTCGTAT-3′ (forward), 5′- AGCCTTCTCCATGGTGGTGAAGAC-3′ (reverse).

### 4.8. Small Interfering RNA

For silencing PS-1 protein expression, a unique siRNA inhibiting human PS-1 (s111) and negative-control siRNA (4390844) were purchased from Applied Biosystems Instruments (Foster City, CA, USA). For silencing the ERK1 protein expression, a unique siRNA inhibiting human ERK1 (SC-29307) and negative-control siRNA (SC-37007) were purchased from Santa Cruz Biotechnology (Santa Cruz, CA, USA). 5 × 10^5^ U2OS cells and HOS cells were grown in 6 cm cell culture dishes overnight. A total of 150 pmol of PS-1 siRNA was transfected into the cells while using lipofectamine RNAiMAX transfection reagent, according to the manufacturer’s instructions (Invitrogen, Carlsbad, CA, USA). The silencer negative control siRNA, a nonsense siRNA duplex, was used as a control.

### 4.9. Statistical Analysis

For all of the measurements, analysis of variance was followed by one-way analysis of variance (ANOVA) with post hoc Turkey’s HSD tests for more than two groups with equal sample sizes per group. When two groups were compared, the data were analyzed whileusing Student’s *t*-test. Each experiment was performed in triplicate and three independent experiments were performed. *p* values < 0.05 was considered to be statistically significant.

## 5. Conclusions

In conclusion, U2OS and HOS cells-derived PS-1 and the c-Raf–MEK–ERK signaling pathway, not JNK, p38 and PI3K/Akt signaling, may both contribute to cellular invasion and migration. This phenomenon of PS-1′s repression of invasion and migration in U2OS and HOS cells could be activated by tomatidine. Certainly, our work reinforces the idea that tomatidine possesses the suggestive behaviors of anti-metastatic properties in human osteosarcoma cells, which contributes to a better understanding of the mechanism that is responsible for these effects.
